# Exploring the land-use urban heat island nexus under climate change conditions using machine learning approach: A spatio-temporal analysis of remotely sensed data

**DOI:** 10.1016/j.heliyon.2023.e18423

**Published:** 2023-07-20

**Authors:** Priyanka Rao, Patrizia Tassinari, Daniele Torreggiani

**Affiliations:** Department of Agricultural and Food Sciences, University of Bologna, 40126, Bologna, Italy

**Keywords:** Spatio-temporal, Built-up index, Google earth engine, Vegetation index, Surface UHI intensity, Machine learning

## Abstract

Urbanization strongly correlates with land use land cover (LULC) dynamics, which further links to changes in land surface temperature (LST) & urban heat island (UHI) intensity. Each LULC type influences UHI differently with changing climate, therefore knowing this impact & connection is critical. To understand such relations, long temporal studies using remote sensing data play promising role by analysing the trend with continuity over vast area. Therefore, this study is aimed at machine learning centred spatio-temporal analysis of LST and land use indices to identify their intra-urban interaction during 1991–2021 (summer) in Imola city (specifically representing small urban environment) using Landsat-5/8 imageries. It was found that LST in 2021 increased by 38.36% from 1991, whereas average Normalised Difference Built-up Index (NDBI) increased by 43.75%, associating with increased thermal stress area evaluated using ecological evaluation index. Major LULC transformations included green area into agricultural arable-land and built-up. Finally, the modelled output shows that built-up & vegetation index have strongly impacted LST. This study, help to understand the relative impact of land-use dynamics on LST at intra-urban level specifically with respect to the small urban settings. Further assisting in designing and regenerating urban contexts with stable configuration, considering sustainability and liveable climate, for benefit of health of public and fragile population in particular.

## Graphical abstract

**Figure:**
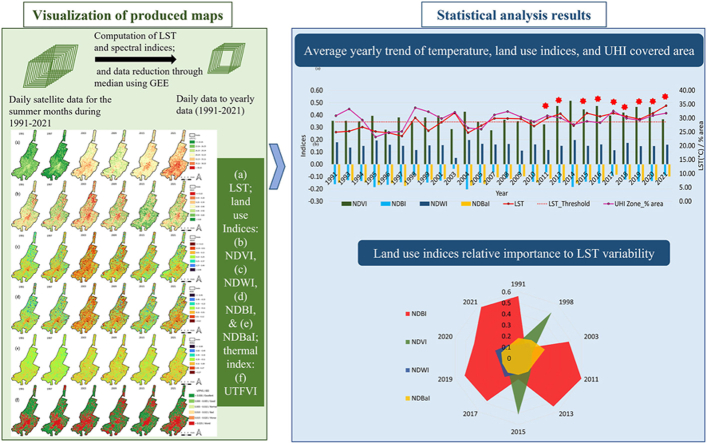

## 1 Introduction

Urbanization’s rapid global acceleration poses a significant challenge in adapting urban areas to climate change. The World Meteorological Organization (WMO) report [1] confirmed that the planet is consistently warming due to human activities, leading to substantial changes in the earth's surface, atmosphere, and water systems. These changes have far-reaching consequences for sustainable growth and ecosystems. The particular concern is the urban heat island (UHI) effect, where urban areas experience higher temperatures compared to nearby rural areas due to urbanization. The rise in urban temperature caused by urbanization [2] has profound implications for public health [3], energy consumption [4], greenhouse gas emission [5], and heat generated by human activity in cities [6]. However, accurately analysing temperature deviations in urban areas has become uncertain, as urban regions also influence nearby rural areas, making it challenging to establish a suitable reference point [7]. To address this, it is crucial to comprehend temperature variability within the urban areas of a city, focusing on the specific climatic modifications and human-induced changes caused by urbanization over the last decades. By understanding these dynamics, we can mitigate the harmful impacts of excessive urban heating [8,9].

Urban areas undergo warming due to the replacement of natural land cover with built-up surfaces, which impacts solar radiation absorption, shade availability, heat and moisture exchange between land and atmosphere, leading to changes in local climate conditions and temperature intensification within cities [10]. Land surface temperature (LST) plays a vital role in understanding the interaction between the Earth's surface and the atmosphere [11]. While several studies have examined the relationship between land use land cover (LULC) properties and LST [[12], [13], [14]], the understanding of the LULC-LST connection in complex small urban areas over longer temporal periods comprehending climate change perspective remains limited. Investigating the influence of land cover changes on climate is essential, as it significantly impacts quality of life, including human health and safety, through its effect on LST [15,16]. To capture localized impacts of land use changes and associated temperature variations, it is necessary to examine the relationship between LST distribution and land covers at the local level over a long period, which previous studies on broader regional or global scales may not adequately represent [13,14]. Despite the increasing research on Surface Urban Heat Island (SUHI) and the identification of various contributing factors, data and methodological limitations hinder the applications of SUHI research [17]. Therefore, it is crucial to compare SUHI intensity estimation approaches to select the most suitable one based on research objectives. Additionally, classifying cities or areas based on ecological comfort is important, leading to the development of different indices such as the Urban Thermal Field Variance Index (UTFVI), which directly relates to LST and plays a significant role in the UHI phenomenon [18].

Land use indices namely normalised difference built-up index (NDBI), normalised difference water index (NDWI), normalised difference vegetation index (NDVI) and normalised difference bareness index (NDBaI) are widely used to differentiate between the built-up, water, vegetation, and soil respectively. The effect of land cover on surface temperature has been compared over different cities and climate zones evaluating the urban area using either simple linear regression approach or considering a short temporal period [13,15,16,19], thereby usually lacking to either to capture complex linear and non-linear relations or changing trend over a longer period. While it is generally understood that urban areas tend to have higher LSTs than rural areas due to UHI effect, the extent to which specific land covers contribute to this phenomenon is not well understood. For instance, the extent to which impervious surfaces such as concrete and asphalt versus vegetation loss contribute to the UHI effect is not well understood.

The goal of this study is to understand how different land use and land cover types affect surface temperature in a unique urban setting. Using detailed remote sensing data over an extended period, we investigate the relationship between land cover and surface heat in the Emilia Romagna region of Italy. This research is one of the first providing insights into surface urban heat and land cover properties, typically in a small & complex urban environment. By employing a machine learning (ML) model, we analyse the temporal dynamics and characteristics of land use. This study aims to uncover the factors contributing to UHI formation and provide quantitative insights into surface urban heat and LULC properties, aiding in the development of effective strategies for mitigating local UHI issues. The specific objectives of this research include: (i) Analysing trends of LST, LULC indices, and UTFVI; (ii) Examining LULC patterns and changes from 1994 to 2017, comparing two approaches for estimating SUHII and assessing its variations over time; (iii) Assessing the relative contribution of land cover drivers to surface temperature dynamics using machine learning.

The paper is structured as follows: Section 2 materials and methods, which includes subsections on the study area, materials used, and the methodology framework. The results are presented in Section 3 and discussed in Section 4. Conclusions are presented in Section 5.

## 2 Materials and methods

### 2.1 Study area

**Fig. 1:**
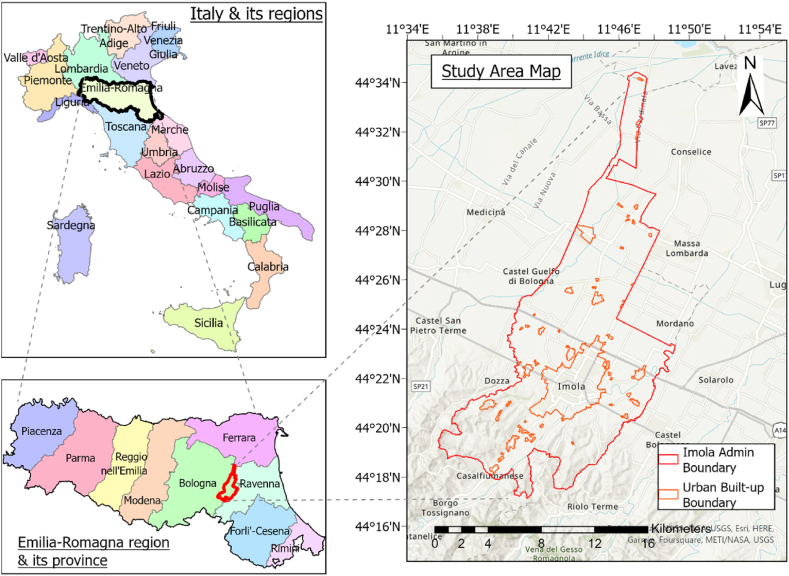
Study Area Map (Left: Top key map showing Italy & its regions, and bottom key map showing Emilia-Romagna & its province; Right: Study area map showing urban built-up area boundaries in 2011).

This study focuses on the Imola municipality area (Fig. 1) in the Emilia-Romagna region of northern Italy. Imola serves as the gateway to the historic region of Romagna and is characterized by a densely populated urban center surrounded by agricultural land, semi-natural areas, and forests. As the second largest municipality in the province of Bologna, Imola experiences a subhumid and temperate climate classified as Cfa according to the Koppen–Geiger climate classification [20]. During the summer season, the air temperature in Imola ranges from 28.1 °C to 31 °C (maximum), 16.9 °C–19.4 °C (minimum), and 22.8 °C–25.3 °C (average) based on data trends from 1991 to 2021 [21]. Italy has witnessed a rapid average temperature increase of 1 °C per year, surpassing the global average, particularly in the past two decades [22]. With a notable rise observed during spring and summer. Relative humidity ranges from 54% to 59%, while summer precipitation amounts to 57 mm–68 mm [21].

Furthermore, based on the several published reports, Italy is considered a country in the European union with the highest population of old age people in year 2021 with 23.5% population aged above 65 years [23], and declining birth rate [24]. Moreover, Emilia-Romagna region of Italy consists a large proportion of elderly people and significant proportion of infants population [25] along with being one of the highly populated regions of northern and central Italy [26]. The province of Bologna has experienced a considerable change from 2018 to 2021 in several demographic factors, for example, birth rate (from 7.4 to 6.9), death rate (from 11.5 to 12.7), natural balance (from −4.1 to −5.8), growth rate (from 4.9 to 0.1), and life expectancy at the age of 65 (from 21.4 to 20.8) [27]. The region is highly urbanised, and its major economy is based on the construction, trade and manufacturing industries [28].

### 2.2 Materials used

The temporal data from Landsat 5 (1991–2011) and Landsat 8 (2013–2021) were utilized to calculate LST and land use indices (see Table 1) specifically for the summer months. Landsat satellites pass the equator at around 10:00 a.m. ( ± 15 min) local time, with a repeat cycle of 16 days. Hence, Landsat data from the summer months between 1991 and 2021 were included in the analysis, excluding years (1992, 2000, 2006, and 2012) with data unavailability, heavy cloud cover or unsuitable atmospheric conditions. For the preparation of land use change matrix and determination of SUHII levels, regional land use maps were employed. To assess the yearly patterns of LST in relation to different LULC types, specific indices were computed for each year. The computation of these indices, along with LST, for a 31-year period during the summer months resulted in a substantial volume of data. To manage this data, including multiple bands for each year, Google Earth Engine (GEE) was utilized as a suitable alternative. GEE facilitated access to processed satellite data through a cloud platform, allowing for the calculation of LST and other indices using appropriate algorithms and spectral band combinations.

**Table 1:** Detailed specifications of datasets used.

Variable/specification	LST	NDVI	NDBI	NDWI	NDBaI	Regional Temporal LULC maps
**Data and time period**	Months: June, July, and August; Year and Data type: Landsat 5 (TM) for 1991 to 2011 & Landsat 8 (OLI/TIRS) for 2013 to 2021					1994, 2003, 2008, 2011, 2014, and 2017
**Landsat 5 bands**	Band no. 2, 3, 4, 6	Band no. 4, 3	Band no. 5, 4	Band no. 2, 4	Band no. 5, 6	N/A
**Landsat 8 bands**	Band no. 3, 4, 5, 10	Band no. 5, 4	Band no. 6, 5	Band no. 3, 5	Band no. 6, 10	
**Method & formula used**	Mono Window Algorithm	$\frac{NIR-Red}{NIR+Red}$	$\frac{SWIR1-NIR}{SWIR1+NIR}$	$\frac{NIR-SWIR}{NIR+SWIR}$	$\frac{SWIR1-TIR}{SWIR1+TIR}$	N/A
**Resolution**	30 m					1.5 ha
**References/Source**	[29]	[30]	[31]	[32]	[33]	[34]

The regional geoportal of Emilia-Romagna region provides land use maps at a spatial resolution of 1.5 ha from 1976 to 2017. To align with the study period from 1991 to 2021, the downloaded LULC maps include those from 1994, 2003, 2008, 2011, 2014, and 2017.

### 2.3 Methods

**Fig. 2:**
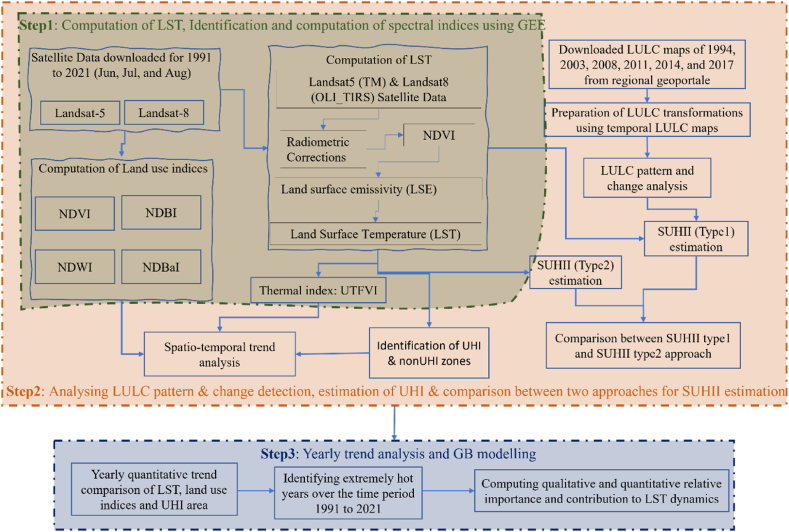
Detailed work flow of implemented methodology.

The study employed three main steps (Fig. 2). First step includes computation and downloading of LST & spectral indices (NDVI, NDBI, NDWI, NDBaI) using GEE, and LULC maps from reginal geoportal. GEE has been used to compute LST and land use indices derived using spectral bands of Landsat data, while thermal index has been calculated using QGIS 3.16.0. The second step consists of spatio-temporal trend analysis of LST with land use indices and LULC changes. Further, SUHII has been calculated using two different methods and compared based on the extent of coverage within different SUHII ranges. Finally, the relative importance and contribution of LULC types as driving factors for LST were evaluated using the GBR model and visualized using SHAP (SHapley Additive exPlanations). Following is the detailed explanation to the methodology for each step.

#### 2.3.1 Computation of LST, spectral indices, and UTFVI

The GEE platform utilized to compute spectral indices and LST to minimize processing time and storage concerns for the extensive 31-year dataset. Cloud-free Landsat-5 and Landsat-8 images, with a spatial resolution of 30 m, were employed. It is worth noting that some pixel values may exhibit extreme temperature peaks and other indices due to the focus on the summer season. To ensure the preservation of variability, calculating the mean of all values for each year may result in the loss of important information from the final yearly spatial product. Consequently, the median value was estimated for each pixel, merging the outcomes from temporal observations within summer months per year to create an annual summer observation. The resulting data was downloaded in geotiff format for further analysis. UTFVI (thermal index) was computed based on LST using GIS.

Despite the sensor differences between Landsat 5 and Landsat 8 satellites, the presence of similar bands with comparable wavelengths allows for their combination. This approach harnesses the strengths of both sensors while mitigating their weaknesses. Landsat 5 data offers a longer time series of LST measurements, while Landsat 8 data provides enhanced spatial information and improved accuracy through additional spectral bands. By combining data from both sensors, a more comprehensive understanding of LST dynamics across time and space can be achieved. To retrieve LST, 30 m resampled thermal infrared images from both Landsat missions were utilized, employing the mono window algorithm (MWA).

##### 2.3.1.1 LST computation using GEE platform

The importance of LST computation and its relation with UHI has been observed in recent research works [12,35,36]. In the process of computation of LST, conversion of digital number (DN) of TIR band to spectral radiance is the first step. To do so, Landsat-5 and Landsat-8 have different algorithm, which are illustrated in equation (1) [37] & equation (2) [38] respectively.

(1) L=0.553*(DN−1)+1.2378

(2) L=0.0003342*DN+0.1003

For thermal infrared band, atmospheric correction has been applied as suggested by Ref. [29]. Further surface radiance has been calculated based on the atmospheric correction (equation (3)).

(3) $$L_{S}=\frac{L-L_{U}+Ʈ*\left(1-\varepsilon \right)*L_{D}}{Ʈ*\varepsilon }$$

Sobrino et al. [39] described surface reflectance as $L_{S}$; L as the top of atmospheric radiance for TIR band; upwelling radiance as $L_{U}$; downwelling radiance as $L_{D}$; atmospheric transmission has been denoted by Ʈ; while ε as the emissivity which has been calculated as f (NDVI) for each pixel and proportion of vegetation ($P_{V}$). The detailed equations have been described below in equation (4):

(4) ε=0.979−0.035*ρ,(NDVI<0.2)

$$\varepsilon =0.986+0.004*\;P_{V},\left(0.2\leq NDVI\leq 0.5\right)$$

ε=0.99,(NDVI>0.5)

where, ρ is the reflectance of the band with red spectral range. NDVI is used to estimate emissivity which has been calculated using reflectance ratio of near infra-red (NIR) and red band, as described below in equation (5), and equation (6) suggests formula to calculate $P_{V}$ [40,41].

(5) $$NDVI=\frac{\left(NIR-R\right)}{\left(NIR+R\right)}$$

(6) $$P_{V}={\left(\frac{NDVI-{NDVI}_{\min}}{{NDVI}_{\max}-{NDVI}_{\min}}\right)}^{2}$$

where, ${NDVI}_{\min}$ and ${NDVI}_{\max}$ are the values indicating minimum and maximum respectively. The final step of LST calculation is mentioned in equation (7) [29].

(7) $$LST\;\left({}^{\circ}C\right)=\left(\frac{K_{1}}{\ln\left(\frac{K_{2}}{L_{S}}+1\right)}\right)-273.15$$

The calculated LST has been used to assess the spatio-temporal variation of surface temperature. Additional thermal index named UTFVI has been estimated based on LST to comprehensively investigate the impact of surface temperature on urban climate and identify areas within the city with higher temperature ranges [42].

##### 2.3.1.2 Urban Thermal Field Variance Index (UTFVI)

A substantial area of lush greenery on land contributes to comfortable and thermally stress-free environment by reducing temperature. UTFVI has been used to assess ecological comfort based on ecological evaluation index (EEI), as lower UTFVI value indicates a higher level of EEI. UTFVI values were categorized into six levels based on EEI: excellent, good, normal, bad, worse, and worst. These categories were determined by predefined intervals: <0.000, 0.000–0.005, 0.005–0.010, 0.010–0.015, 0.015–0.020, and >0.020. UTFVI algorithm is described in Equation (8).

(8) $$UTFVI=\frac{\Delta T}{T_{M}}=\frac{T_{i}-T_{M}}{T_{M}}$$

where, $T_{i}$ = pixel LST; and $T_{M}$ = average temperature of study area [43].

##### 2.3.1.3 Computation of land use indices

The temperature of urban and non-urban areas is influenced by the type of land cover class in vicinity. Hence, in addition to LULC classification, researchers have utilized various land use indices derived from remotely sensed data to examine annual landscape changes [44,45]. Land use indices NDVI, NDWI, NDBI, and NDBaI, have been calculated to analyse the spatio-temporal dynamics of vegetation, water/surface moisture, built-up, and bare land/soil respectively. The spectral band combinations used for computing these indices are provided in Table 1. The normalised difference spectral indices range from −1.0 to +1.0, with higher positive values indicating vegetation, water surfaces and high surface moisture, built-up areas, and bare land or soil, respectively. Vegetation is typically denoted by NDVI values above 0.2, while NDWI values above 0.0 represent water and also vegetation above 0.05. NDBI values between 0.10 and 0.25 indicate the presence of built-up areas, and NDBaI values above 0 indicate bareness [46]. These land use indices are employed to identify different LULC classes (e.g., vegetation, built-up areas) by determining suitable threshold values.

#### 2.3.2 Analysing LULC pattern & change detection, estimation of UHI zones & comparison between two approaches for SUHII estimation

##### 2.3.2.1 Analysis of LULC spatio-temporal pattern and change detection

The LULC maps acquired from regional geoportal of Emilia Romagna region for 1994, 2003, 2008, 2011, 2014, and 2017 were reclassified into 6 major classes based on their impact and feedback to increasing temperature. The new classes are: Water & wetlands, Forests, Permanent crops & green areas, Agricultural arable land, Built-up, and Open/waste land (supplementary table s1). Further, LULC pattern and change dynamics have been analyzed using detailed LULC change matrix showing quantitative change of one specific class into another.

##### 2.3.2.2 Identification of UHI and nonUHI based on LST

UHI and nonUHI zones have been estimated using LST (equations (9), (10))), and were delineated and mapped using GIS.

(9) UHI = LST > μ + 0.5 *δ

(10) NonUHI = 0 < LST≤μ + 0.5 *δ

where, μ is mean LST of the study area; and δ is the standard deviation [47].

##### 2.3.2.3 Computation of SUHII using two different approaches and their comparison

In this study, we computed SUHII using two commonly utilized methods. The SUHII calculation involved averaging the LST of green spaces such as forests and other vegetated areas, and subtracting it from temperature of study area [48] (equation (11)), and referred as SUHII type1 approach. Negative temperature values obtained were assigned to non-built-up category. The None SUHII category comprised pixels with value = 0, while values > 0 were classified into five intensity levels: low (0–2), moderate (2–4), high (4–6), very high (6–8), and extremely high (>8). In an alternative approach, UHI threshold was deducted from study area (equation (12)). The resultant values were then divided into five intensity levels, similar to SUHII type1 method. This equation considers temperature variations across study area and establishes a threshold value for distinguishing UHI and non-UHI regions, regardless of surface characteristics (as determined in equation (9)). The outcomes obtained from this process were referred as the SUHII type2 approach.

(11) $$SUHII\;Type1={LST}_{i}-{LST}_{Green\;spaces}$$

(12) $$SUHII\;Type2={LST-SUHI}_{threshold}$$

where, i is the LST at any pixel, and SUHI threshold is UHI threshold estimated in equation (9).

Both approaches were compared in terms of spatial extent of each SUHII level starting from low to extremely high levels. Statistical comparisons were performed to assess the correlation between percentage area covered by each level for both approaches, and quantified variations by calculating RMSE, MAE, and standard deviation.

#### 2.3.3 Quantitative yearly trend analysis of LST, land use indices, UHI zone, & their cumulative linear correlation

The study examines the annual variations in LST and land use indices during the summer season. By calculating a threshold value using the average temperature across studied time-period, years crossing calculated threshold, were marked as exceptionally hot years.

#### 2.3.4 Quantitative and qualitative contribution of land cover types towards LST

The scikit-learn Python package was used to implement ML techniques and explore the relationship between land cover variables and summer season LST variation over multiple years. Specifically, gradient boosting (GB) regression model that constructs decision trees sequentially to minimize prediction errors, was employed [49]. GB regression has been widely effective in various fields due to its boosting method and shallow trees, which prevent overfitting [9,50]. Each decision tree in the ensemble, predicts LST based on a subset of features, with trees trained sequentially to improve on errors made by previous trees. This iterative process continues until the model achieves satisfactory accuracy. The impact of independent variables such as NDVI, NDBI, NDBaI, and NDWI on LST variation (target variable) was assessed using GB regression. During training, the GBR model assigns weights to features, indicating their importance in predicting LST. These weights are determined based on each feature's contribution to the overall model performance. Hyperparameters were optimized to obtain the best-fit model with high accuracy. The model was trained and tested using an 80:20 ratio. Model accuracy and precision were evaluated using adjusted R^2^ and RMSE metrics, respectively (Fig. 8). Variable importance in the GB model signifies each variable's contribution to reducing the model fit's variance. To analyse the impact of climate change, the model was used to assess the importance of each explanatory variable for years with high temperatures. Variable importance was extracted only if the model demonstrated satisfactory prediction accuracy on the corresponding test dataset (adjusted R^2^ ≥ 0.7). To ensure robustness to random sampling, the entire process was repeated multiple times (8−10). The importance scores for selected hot years were plotted using radar charts, and the results were further examined using SHAP variable plots.

SHAP variable plots offer an intuitive visualization of the influence of each land use index on the model's output. They serve as a global interpretation of model, revealing the contribution of each feature to the model's output for individual data points. Consequently, these plots aid in explaining the model's predictions and instilling confidence in its performance. Ultimately, they facilitate a comprehensive understanding of the GBR model's behaviour concerning LST and land cover indices, augmenting interpretability and providing valuable insights into the prediction process.

## 3 Results

### 3.1 LST, UTFVI and land use indices

**Fig. 3:**
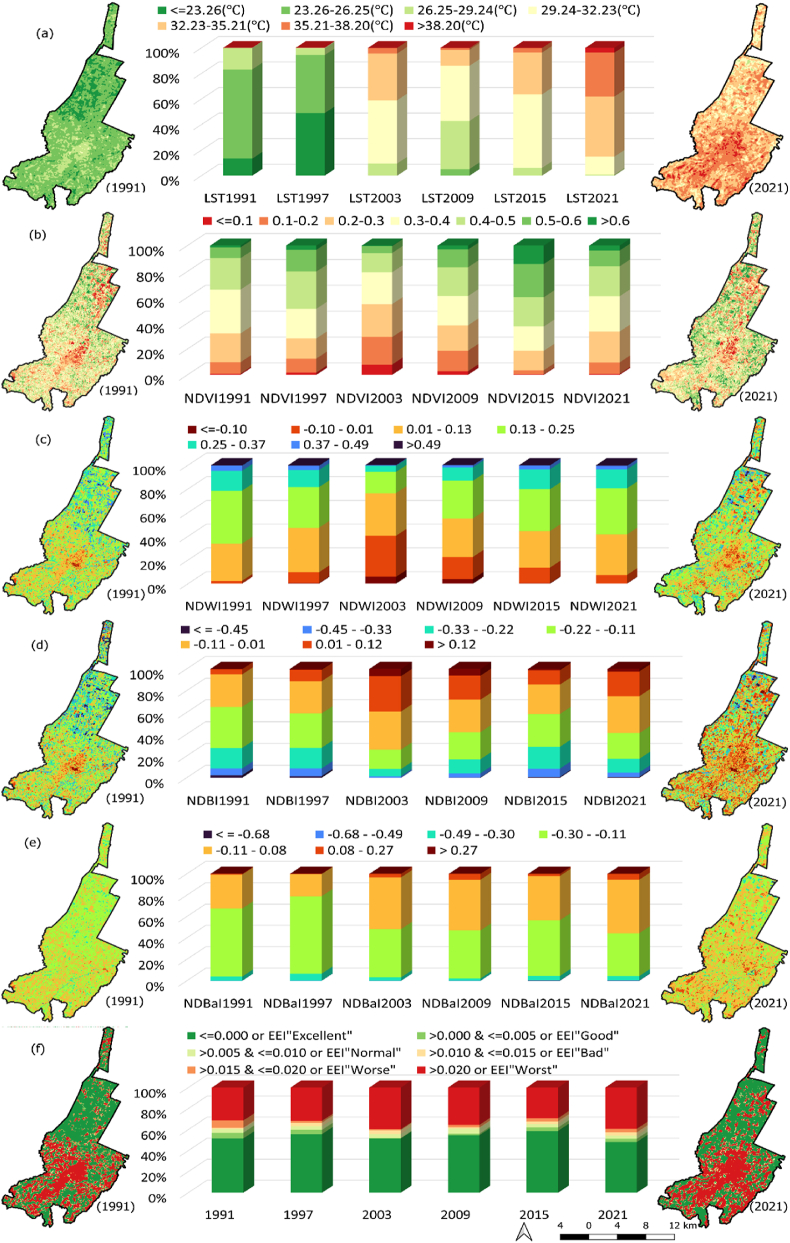
Spatial distribution and temporal dynamics of (a) LST; (b) NDVI; (c) NDWI; (d) NDBI; (e) NDBaI; (f) UTFVI; for 1991, 1997, 2003, 2009, 2015, and 2021, where spatial distribution map has been shown for 1991 and 2021 and temporal dynamics graph of area under different LST and indices range has been illustrated.

Fig. 3(a–f) depicts the spatial variability and area dynamics of various indices (LST, NDVI, NDWI, NDBI, NDBaI, and UTFVI, respectively). To visualize the spatio-temporal surface temperature variation, temperature values were divided into 7 equal intervals, ranging from≤23.26 °C to >38.20 °C. Notably, the central region of the Imola municipality experienced a significant temperature increase from 1991 to 2021 (Fig. 3a). The maps and graphs display a clear year-by-year expansion of the area under higher temperature levels. Since 1991, the average and maximum recorded temperatures have risen by approximately 10 °C, while the minimum temperature has increased by around 7.40 °C (Supplementary table s2 details out the annual statistics).

In this study, four land use indices (NDVI, NDWI, NDBI, and NDBaI) were calculated using Landsat-5 and Landsat-8 satellite data. The spatial distribution of these indices (Fig. 3) shows that the city center mainly consists of built-up areas, while the surrounding region comprises agricultural land and vegetation. NDVI shows fluctuating trend with a decline in 2003 and an increased area in both higher and lower NDVI ranges in 2021. NDWI follows a similar pattern to NDVI. Mean NDVI in 1991 and 2021 was 0.35 and 0.37, respectively, and mean NDWI was 0.18 and 0.16. NDBI and NDBaI indicate an increasing trend, with higher values suggesting an expansion of built-up and bare land (More detailed statistics can be found in supplementary table s3).

The spatial illustration of UTFVI shows that majorly it is covered by two EEI categories i.e., excellent (UTFVI<0.000) and worst (UTFVI>0.020) (Fig. 3f). The spatio-temporal trend of EEI shows that built-up area (spread in center and southern part), falls under worst category. As we track from excellent to worst EEI range, the percentage area under worst category has changed from 31.35% to 39.29%, whereas for excellent category from 51.60% to 48.15% during 1991–2021. (For detailed yearly statistics of EEI, see supplementary table s4).

### 3.2 LULC pattern, change detection, and SUHII analysis

**Fig. 4:**
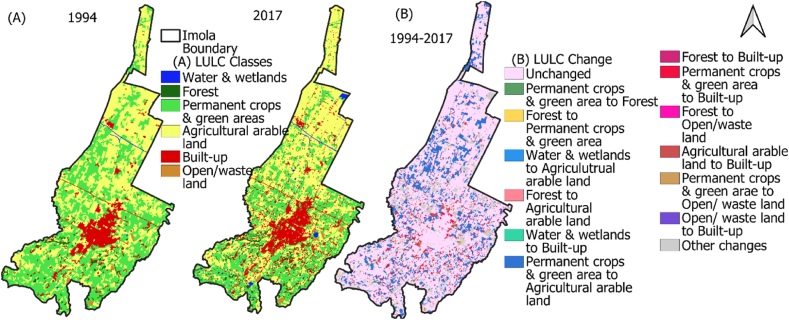
(A) Land use land cover in 1994 & 2017, (B) spatio-temporal LULC change dynamics for Imola during 1994–2017.

LULC spatial distribution indicates concentrated urban built-up areas in the center, with small suburbs nearby (Fig. 4A). From 1994 to 2017 the built-up area increased significantly, particularly in north-south direction. Southern Imola features permanent crops, vegetation, and forests, while the north is dominated by agricultural arable land. Open/waste land and water-bodies showed minimal change. Fig. 4B illustrates the dynamic transformation of LULC classes between 1994 and 2017, with permanent crops and green areas converting to built-up and then agricultural arable land. The LULC change matrix (Table 2) reveals increased agricultural arable land (44.46 sq.km. to 47.46 sq.km.) and built-up areas (8.94 sq.km. to 14.63 sq.km.) but decreased permanent crops and green areas (44.44 sq.km. to 33.67 sq.km.) from 1994 to 2017. Other LULC classes remained relatively stable.

**Table 2:** LULC change matrix from 1994 to 2017, where A, B, C, D, E, and F represents: water & wetlands, forest, permanent crops & green areas, agricultural arable land, built-up, and open/waste land area. LULC class and its percentage area in each row of the matrix represents 1994, while each column for 2017. Diagonal values show unchanged percentage area.

**Fig. 5:**
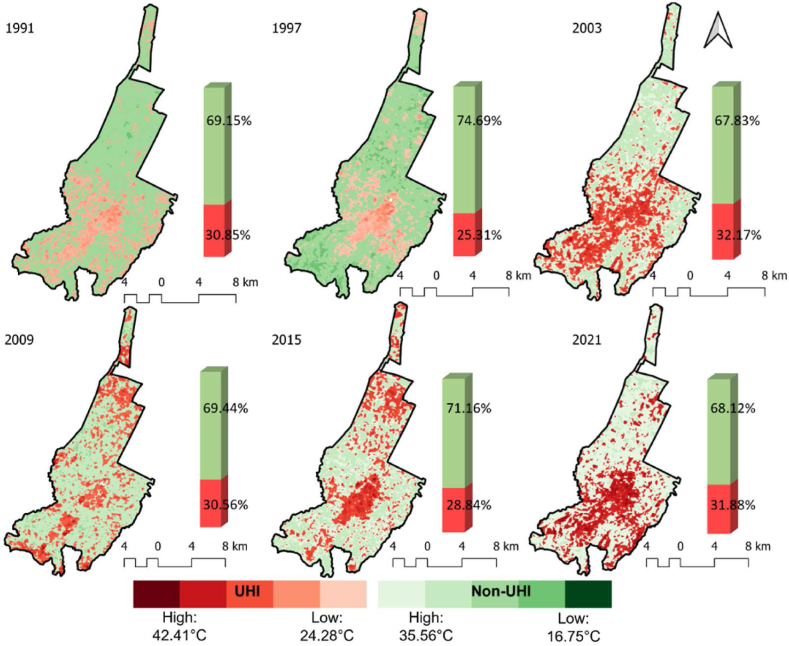
Spatio-temporal dynamics of UHI and Non-UHI zonation during summer season from 1991 to 2021.

**Fig. 6:**
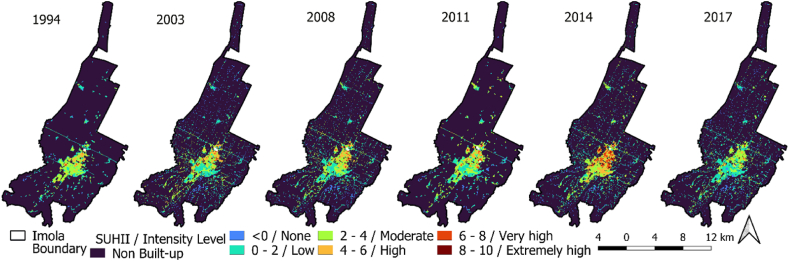
Spatio-temporal dynamics of SUHII type1 for Imola during 1994–2017.

The UHI zone delineated based on LST, predominantly covered core built-up area. It expanded northward from 1991 to 1997 (Fig. 5) and shifted southward in 2003, becoming more dispersed by 2009. In 2015, UHI zone centred in the north, spreading southward in 2021. The UHI zone follows a similar trend to the worst EEI zone. Furthermore, the intensity of SUHI is categorized into six levels. Fig. 6 displays the spatial variation of SUHII calculated based on type1. Higher levels (high, very high, and extremely high) of SUHII are spotted in the northern part of core built-up fabric. Amongst different SUHII level, maximum percentage of area has been covered either under low (0–2) or moderate (2–4) intensity levels. Major changes have been observed by area under low SUHII level with an increase from 3.89% in 1994 to 8.10% in 2017.

The statistical comparison of SUHII levels using type1 and type2 approaches reveal significant findings. The type2 method, utilizing UHI threshold and temperature at each pixel, identifies high heat areas irrespective of built-up or non-built-up boundaries. In contrast, type1 SUHII approach incorporates LULC data and LST, focusing solely on built-up regions and excluding non-built-up areas. Consequently, the type2 approach encompasses a larger low-SUHII area compared to type1 due to the latter's emphasis on differentiating built-up and non-built-up regions using LULC patterns. The type1 approach ensures accurate SUHII estimation in regions with substantial human-induced modifications (Fig. 5 and Table 3). Conversely, the type2 approach covers broader range of surfaces without explicitly distinguishing between built-up and non-built-up areas based on physical characteristics. This comparison enhances our understanding of the limitations and potential advantages of these approaches, highlighting the importance of LULC in accurately estimating lower SUHII levels based on surface characteristics.

**Table 3:** Statistical analysis of SUHII calculation Type1 and Type2 approach, where Type1 is using LULC and LST map, and Type2 is using UHI threshold value and LST.

Year	Analysis excluding low SUHII level				Analysis including low SUHII level			
	Correlation	RMSE	MAE	St. Dev.	Correlation	RMSE	MAE	St. Dev.
**1994**	0.99	0.28	0.19	1.77	0.66	9.64	4.46	7.66
**2003**	0.97	1.23	0.83	2.5	0.89	8.73	4.54	7.78
**2008**	0.97	1.02	0.73	2.4	0.9	8.78	4.49	7.98
**2011**	0.98	1.08	0.74	2.52	0.77	8.77	4.49	7.32
**2014**	0.96	0.7	0.52	1.78	0.79	7.57	3.79	6.6
**2017**	1	0.74	0.46	1.53	0.92	9.81	4.74	9.33

### 3.3 Temporal trend and correlation analysis for LST, UHI area & land use indices

**Fig. 7:**
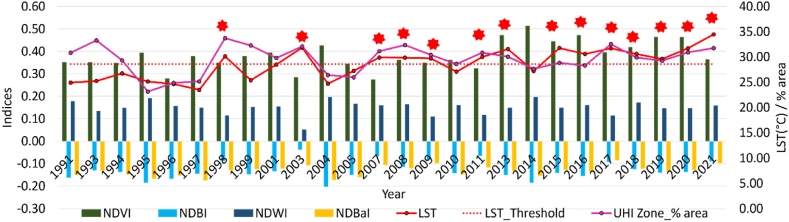
Land use indices, LST and UHI area from 1991 to 202, where LST threshold is average LST from 1991 to 2021. Red stars highlight extreme hot years with respect to average temperature. (For interpretation of the references to color in this figure legend, the reader is referred to the Web version of this article.)

Fig. 7 depicts the temporal dynamics between land use indices and LST, illustrating yearly fluctuations from 1991 to 2021. Notably, during 1998, 2003, 2007–09, 2011, 2013, and 2015–21, summer temperatures exceeded the calculated threshold (28.62 °C i.e., average LST over 31years). These years, denoted by red stars in the graph, are identified as periods of extreme heat.

**Fig. 8:**
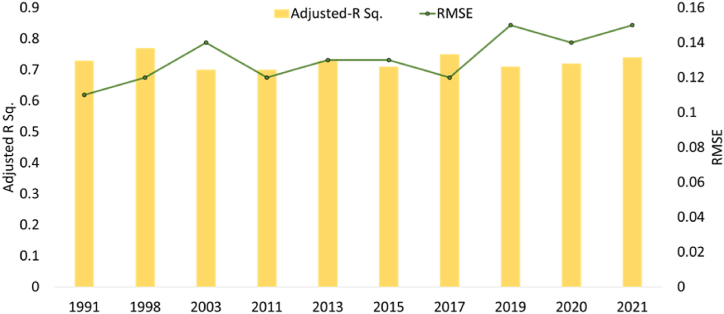
Model accuracy (explanatory potential of all indices on LST) and precision/model fit illustrated by RMSE and adjusted R^2^ respectively for the identified extremely hot years during 1991–2021. The results are statistically significant at 0.05 alpha value.

The bivariate correlation matrix (Table 4) revealed a range from −1 to +1, indicating strong negative to positive correlations. LST exhibited a weak negative correlation with NDVI and a relatively strong negative correlation with NDWI. Conversely, NDBI and NDBaI displayed a very strong positive correlation with LST. UHI area demonstrated very strong positive correlation with NDBI and NDBaI, while weak negative and strongly negative correlations with NDVI and NDWI, respectively. LST exhibited a positive correlation with UHI area and a weak correlation with UTFVI. These findings suggest that LST is valuable for UHI-related studies, with direct positive relationships. Additionally, a weak positive bivariate correlation was observed between UTFVI and UHI area, indicating that UHI area alone cannot explain EEI zonation accurately, emphasizing the need for heat island intensity analysis.

**Table 4:** Correlation matrix using Pearson's correlation (p < 0.05). R values are depicted by numbers against each row with respect to the parameter in respective column. The minimum and maximum limit are −1 to +1.

### 3.4 Contribution of LULC factors to LST dynamics

The hottest years within 1991–2000 and 2001–2010 were 1998 and 2003, respectively. Since 2011, most years have exceeded LST threshold. Hence, we chose alternate years for analysis: 2011, 2013, 2015, 2017, 2019, and 2021, employing the GBR model. Fig. 7 indicates a rise in LST from 2019 to 2020, without significant variations in the indices. Thus, to assess feature importance in such scenarios, we also included 2020.

**Fig. 9:**
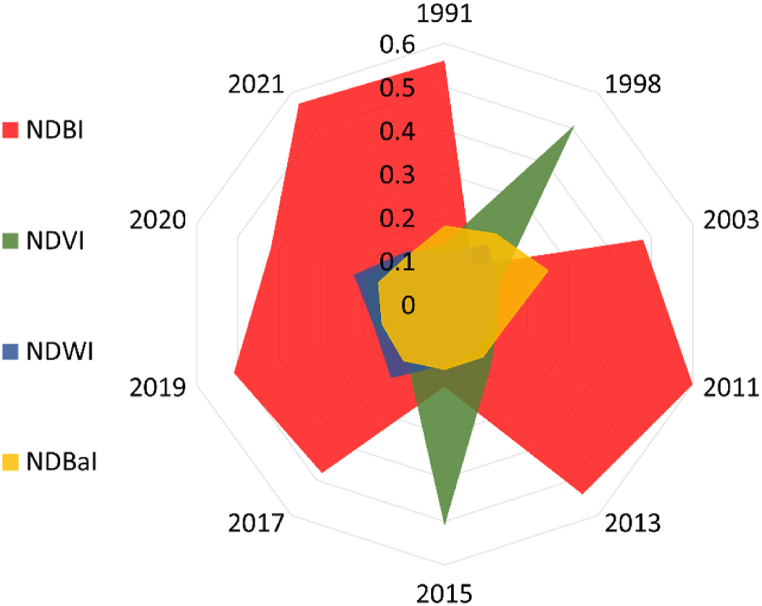
Relative importance of land cover indices in LST variability for the identified extremely hot years.

**Fig. 10:**
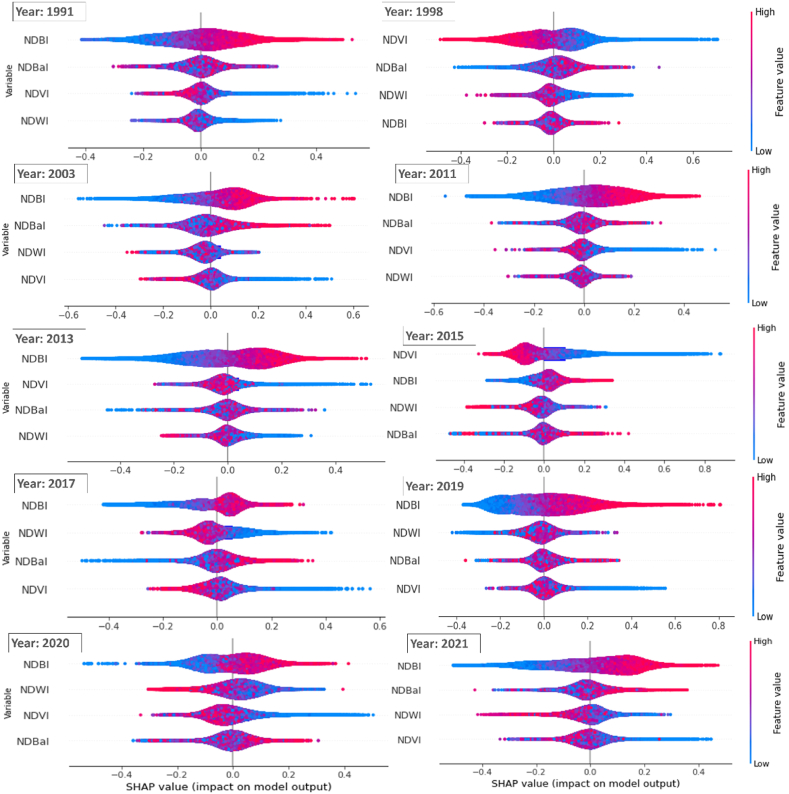
SHAP variable plot for the identified extremely hot years during 1991–2021.

Fig. 8 demonstrates model's accuracy and validation. Fig. 9 displays relative importance of land cover indices compared to LST in different years, determined by the GBR model. During warm periods, NDBI shows the highest impact on LST variability in tropical, temperate, and cold climates, followed by NDVI [9], and similar observation resulted from this analysis. SHAP variable plot (Fig. 10) delivers information related with: (i) Feature importance variables ranked in descending order; (ii) Impact: horizontal position indicates effect of that variable on increased/decreased predicted LST [51]; and Color of plot shows if variable is high (in red) or low (in blue) for that observation.; (iii) Correlation: A high value of “NDBI” has high and positive impact on LST. The “high” comes from the red color, and the “positive” impact is shown on the x-axis. Similarly, “NDVI” is negatively correlated with LST. NDBI consistently emerged as a significant predictor of LST variation throughout the years, while the contributions of other indices varied.

The higher temperatures of summer season led to increased heat absorption and retention by built-up surfaces, resulting in higher LST values. Vegetation, helps regulate temperature in urban areas by absorbing and dissipating heat. Analysis of Fig. 9, Fig. 10 reveals that NDVI primarily contributed to LST increase in 1998 and 2015, emphasizing the importance of vegetation loss in driving higher temperatures. Overall, the combination of NDVI and NDBI provides valuable insights into factors influencing LST variation in urban areas.

## 4 Discussions

This study analyses long-term trends in urban land cover characteristics during the summer season and explores their impact on intra-urban surface temperatures in context of climate change. To comprehend the relationship between land cover types and temperature variability in urban areas, it is crucial to consider changing climate scenarios. Previous research has utilized linear regression over extended periods or compared climate zones using short-term data [9,52]. By examining Imola, a compact municipality with unique land use patterns including historical sites, industrial areas, residential neighbourhoods, and natural landscapes, we aimed to understand the interplay between land cover and surface temperature. This investigation sheds light on specific dynamics of land use, thermal patterns, and microclimates in smaller urban settings, which have received limited attention previously. Imola, like many other urban areas, experiences the UHI effect, leading to elevated temperatures in urban and surroundings compared to rural area. By examining the relationship between land cover and surface temperature in Imola, we can identify the UHI drivers and develop targeted mitigation strategies. The changing trends in land cover attributes and temperature are depicted in Fig. 3, while Fig. 4A and 4B illustrates corresponding changes in land use. These changes subsequently influence UHI intensity (Fig. 5), the distribution of UHI zones (Fig. 6), and thermal stress in urban areas (Fig. 3f).

Previous regional and global research have examined the UHI intensity in major cities worldwide using MODIS imagery [52,53]. They discovered that the daytime UHI intensity negatively correlates with differences in vegetation cover and urban-suburban activity [52], and the distribution of UHI intensity is influenced by various factors such as “vegetation, anthropogenic heat releases, built-up intensity, etc.” [53]. However, regardless of climatic conditions, cities generally experience heightened heating, leading to increased discomfort and energy consumption. Consequently, it is vital to focus on the absolute surface temperature variations within the city and their driving forces, rather than solely comparing urban and rural areas based on UHI intensity. Moreover, this study delineates UHI zones (Fig. 5) and compares two UHI estimation approaches (Fig. 6 and Table 3), highlighting the importance of considering the strengths and limitations of each method for reliable results. Researchers should consider the research objective and area characteristics, to determine more suitable method. If the goal is to understand the impact of LULC on UHI effect, Type1 approach is preferable due to its explicit consideration of these factors. However, if the primary focus is comparing UHI magnitudes across different locations without considering underlying physical mechanisms, type2 approach may be more appropriate.

### 4.1 Linear relationship analysis of land cover indices and LST

The urban-suburban surface temperature differences vary for different urban areas based on their surface characteristics and fabric type [9]. It is crucial to evaluate and understand the temperature trend and land cover variability within the urban area, to assess urban overheating and planning corresponding mitigation strategies. The presence of vegetation impacts the balance between latent and sensible heat fluxes, contributing to cooling effect on LST through transpiration [52]. However, our linear regression analysis reveals a weak correlation between NDVI and LST (Table 4), contrary to the expected strong association of higher vegetation cover with lower temperatures [9,35,54], as linear regression may not effectively capture the complex non-linear interactions present in the data. In contrast, the more flexible and adaptive GB model, reveals a negative relationship between NDVI and LST in this case-study. This suggests that as NDVI exceeds a certain threshold, LST tends to decrease. This non-linear pattern is visualized using SHAP variable plot (Fig. 10). Factors such as vegetation types, vegetation stress, or heterogeneous land cover within the study area can contribute to these non-linear patterns. The relationship between vegetation and temperature highlights the crucial role of moisture availability and healthy vegetation in controlling surface heating and cooling effects. In the absence of urban vegetation and irrigation, cities can become a source of sensible heat.

Urban areas exhibit distinct characteristics that contribute to higher daytime surface heat storage. Factors such as lower surface albedo and emissivity, greater heat conductivity, and larger heat capacity play a role in this phenomenon [55,56]. The intensity of radiation trapping increases with the density of built-up structures. NDBI values serve as a useful indicator for identifying densely built urban regions [31]. Table 4 demonstrates a significant positive correlation between NDBI and LST. However, the temporal pattern of NDBI aligns with that of NDVI, as illustrated in Fig. 3d, b, and Fig. 7. This implies a negative relationship between vegetation-induced cooling effects and spatial variations in built-up areas, consistent with findings from other studies [9].

The NDBaI shows unique temporal patterns (Fig. 3e), and its correlation with LST is illustrated in Fig. 7 and Table 4. It indicates soil distribution in urban areas [46]. While previous research has suggested that soil characteristics may explain LST variability broadly [57,58], our study found that NDBaI did not significantly contribute to it. This highlights the importance of considering variables that better represent soil changes in future studies contributing to surface heat. Additionally, it is important to note that UTFVI and UHI, represent different aspects of the urban thermal environment. The weak correlation between UTFVI and LST can be attributed to specific range of values of UTFVI, as it focuses on classifying thermal field variability into distinct zones rather than capturing fine-scale temperature variations, unlike UHI, which indicates the presence or absence of UHI effect. Moreover, linear regression may inadequately depict the intricate interconnections within complex urban setting due to its incapacity to capture any kind of non-linear relationship.

### 4.2 Land cover indices and their cumulative impact on LST

The analysis, using the GBR model, showed that the examined parameters collectively accounted for changes in LST. The impact of land cover variables on surface temperature differs with the changing climate. While NDBI was the main contributor to surface temperature in most years, NDVI became the most significant variable in certain years like 1998 and 2015 (Fig. 9, Fig. 10). Even though NDVI has a relatively lower influence on surface temperature in urban settings, it still plays a crucial role. This study differs from previous ones that relied on linear regression [59,60], or focused on urban-suburban differences instead of intra-urban variability over a long period [16,52,61,62]. The findings are consistent for the summer season, and further research can explore seasonal variability. The study confirms that NDBI and NDVI are significant factors contributing to surface temperature in urban areas with similar settings and climate, aligning with previous studies [9,53]. This highlights the importance of land management decisions, such as urban greening initiatives and limiting built-up areas, to mitigate urban heat impacts in Imola and cities with a similar climate.

### 4.3 Limitations and future work

This study examines urban land cover characteristics and their response to heat during summer months over a long period. The findings have implications for assessing urban overheating and implementing land cover planning to enhance urban thermal environments in similar climate regions. However, the study has limitations, including its focus solely on daytime surface temperatures and lack of consideration for diurnal variability. To expand the study's scope, a global time-series analysis could be conducted to understand the influence of different land variables on temperature across various climate zones and seasons within a climate change scenario. Additionally, future research could explore advanced machine learning techniques to address missing data and employ multiple regression to assess the relationship between temperature, SUHII, and independent variables within complex urban environments.

## 5 Conclusion

This study utilizes advanced GEE platform to estimate LST and land use indices using long-term earth observation data, focusing on intra-urban trends in small urban area. We aimed to enhance the understanding of the impact of land cover changes on LST dynamics during the summer season in complex urban settings with a temperate and subhumid climate. The transformation of land cover classes reveals a clear relationship between increasing temperature and the shift from permanent crops and green areas to agricultural arable land and ultimately to built-up areas, leading to an expansion of the UHI effect. For estimating localized surface UHI in urban areas, it is crucial to have detailed LULC map in addition to temperature data. However, if the main objective is to compare UHI magnitudes across different locations without considering the underlying physical mechanisms, computing the SUHII based on temperature thresholds is more appropriate. The ML model employed in this study effectively captures the complex relationship between land cover indices and LST, demonstrating the quantitative and qualitative contributions of these indices to LST variability. The results highlight the significant role of NDBI in LST dynamics during the summer season in changing climate scenario, while also acknowledging the considerable importance of NDVI. Thus, the land use-UHI nexus varies over time, influenced by the rate of urbanization and the green-blue to grey ratio of the area.

The study is significant for small municipalities settings like Imola, where urban planning decisions impact the local climate and residents' well-being. The findings offer insights for urban planners, policymakers, and municipal development authorities to guide future development strategies. Maintaining a balance of natural vegetation is crucial in mitigating urban heat. Underutilized land areas can be transformed into green public parks with ponds and wetlands. Emphasizing and promoting policies for conserving natural vegetation, including community forests and water bodies, will contribute to a healthier and safer urban environment, enhancing residents' quality of life.

## Author contribution statement

Conceived and designed the experiments: Priyanka Rao, Daniele Torreggiani.

Performed the experiments: Priyanka Rao.

Analyzed and interpreted the data: Priyanka Rao, Daniele Torreggiani.

Contributed reagents, materials, analysis tools or data: Patrizia Tassinari.

Wrote the paper: Priyanka Rao, Daniele Torreggiani, Patrizia Tassinari.

## Data availability statement

Data will be made available on request.

## Funding statement

The research has been carried out within the PhD project “Big data and "healthy cities": regeneration of urban contexts, green systems and safe and healthy lifestyles”, funded by the Emilia-Romagna Region (Italy) within the Research training projects “Big Data per una regione europea più ecologica, digitale e resiliente” (Fondo POR FSE – Resolution n. 752 of 24/05/2021).

## Declaration of competing interest

The authors declare that they have no known competing financial interests or personal relationships that could have appeared to influence the work reported in this paper
