# Supplementary material for: Exploring the land-use urban heat island nexus under climate change conditions using machine learning approach: A spatio-temporal analysis of remotely sensed data
Source: Heliyon. 2023 Jul 20;9(8):e18423. doi: 10.1016/j.heliyon.2023.e18423 (PMC10391954; doi:10.1016/j.heliyon.2023.e18423)
Supplement: Multimedia component 1 [file mmc1.docx]

**Supplementary Tables**

Table S1 Reclassification of the LULC map downloaded from regional geoportal into new classes based on their feedback to LST

| **Reclassified Class** | **Included Original Classes** |
| --- | --- |
| **Water & wetlands** | Inland & brackish wetlands; aquaculture; canals & waterways; natural basins; artificial reservoirs. |
| **Forests** | Coniferous forests; mixed forests; and broad-leaved forests. |
| **Permanent crops & green areas** | Permanent crops; heterogenous crops; and semi natural areas.  (For example, cultivated poplar groves, scrubland, parks, urban cultivated areas, nurseries, vineyards, orchards, bushes and shrubland, recent afforestation). |
| **Agricultural-arable land** | Arable Land (For example, non-irrigated and irrigated arable land and rice fields). |
| **Built-up** | Artificial surfaces consisting of concrete and paved surfaces.  (For example, residential area, industrial and commercial area, services and hospital settlements, road networks). |
| **Open / waste land** | Mining areas and waste landfills. |

Table S2 Yearly statistical dynamics of the satellite derived land surface temperature (in ℃) for summer season during 1991-2021

| **Year** | **Min. Temp (℃)** | **Max. Temp (℃)** | **Mean Temp (℃)** | **Percentage change in Mean Temp (%) w.r.t. 1991** | | **St Dev (℃)** |
| --- | --- | --- | --- | --- | --- | --- |
| 1991 | 19.99 | 32.44 | 24.92 | Base year | 1.37 | |
| 1993 | 17.39 | 34.30 | 25.25 | 1.32 | 2.04 | |
| 1994 | 22.27 | 35.09 | 26.76 | 7.38 | 1.47 | |
| 1995 | 20.01 | 39.13 | 25.16 | 0.96 | 1.63 | |
| 1996 | 19.61 | 37.03 | 24.67 | -1.00 | 1.57 | |
| 1997 | 17.05 | 33.47 | 23.51 | -5.66 | 1.55 | |
| 1998 | 22.28 | 40.20 | 30.13 | 20.91 | 2.25 | |
| 1999 | 18.27 | 37.11 | 25.42 | 2.01 | 2.46 | |
| 2001 | 21.08 | 39.81 | 28.51 | 14.41 | 2.26 | |
| 2003 | 25.31 | 40.60 | 31.86 | 27.85 | 1.95 | |
| 2004 | 16.75 | 37.47 | 24.72 | -0.80 | 2.04 | |
| 2005 | 21.82 | 39.26 | 27.27 | 9.43 | 1.90 | |
| 2007 | 23.63 | 42.13 | 29.90 | 19.98 | 1.83 | |
| 2008 | 23.31 | 39.81 | 29.86 | 19.82 | 1.95 | |
| 2009 | 22.31 | 40.21 | 29.72 | 19.26 | 2.30 | |
| 2010 | 20.51 | 37.11 | 27.09 | 8.71 | 1.77 | |
| 2011 | 20.90 | 39.82 | 30.01 | 20.42 | 2.01 | |
| 2013 | 24.80 | 41.50 | 31.58 | 26.72 | 2.10 | |
| 2014 | 21.79 | 37.89 | 27.23 | 9.27 | 1.76 | |
| 2015 | 27.10 | 41.40 | 31.78 | 27.53 | 1.77 | |
| 2016 | 24.89 | 40.22 | 30.59 | 22.75 | 1.76 | |
| 2017 | 26.00 | 38.49 | 31.73 | 27.33 | 1.55 | |
| 2018 | 26.10 | 38.94 | 30.60 | 22.80 | 1.74 | |
| 2019 | 24.59 | 38.38 | 29.61 | 18.82 | 1.90 | |
| 2020 | 23.57 | 41.89 | 31.73 | 27.33 | 2.48 | |
| 2021 | 27.38 | 42.41 | 34.48 | 38.36 | 2.14 | |

Table S3 Statistical details of the land use indices cumulative for Jun, Jul, and Aug months during 1991-2021

| **Year** | **NDVI** | | | | **NDBI** | | | | **NDBaI** | | | | **NDWI** | | | |
| --- | --- | --- | --- | --- | --- | --- | --- | --- | --- | --- | --- | --- | --- | --- | --- | --- |
|  | **Min** | **Mean** | **Max** | **St**  **Dev** | **Min** | **Mean** | **Max** | **St**  **Dev** | **Min** | **Mean** | **Max** | **St**  **Dev** | **Min** | **Mean** | **Max** | **St**  **Dev** |
| 1991 | -0.15 | 0.35 | 0.70 | 0.11 | -0.66 | -0.16 | 0.20 | 0.12 | -0.82 | -0.15 | 0.25 | 0.09 | -0.16 | 0.18 | 0.63 | 0.10 |
| 1993 | -0.10 | 0.35 | 0.71 | 0.12 | -0.74 | -0.13 | 0.23 | 0.12 | -0.82 | -0.13 | 0.25 | 0.09 | -0.18 | 0.14 | 0.70 | 0.11 |
| 1994 | -0.16 | 0.35 | 0.69 | 0.12 | -0.67 | -0.14 | 0.52 | 0.13 | -0.83 | -0.15 | 0.74 | 0.10 | -0.20 | 0.15 | 0.63 | 0.11 |
| 1995 | -0.14 | 0.39 | 0.71 | 0.12 | -0.79 | -0.19 | 0.50 | 0.14 | -0.87 | -0.17 | 0.74 | 0.10 | -0.21 | 0.19 | 0.72 | 0.11 |
| 1996 | -0.09 | 0.28 | 0.68 | 0.10 | -0.75 | -0.17 | 0.54 | 0.12 | -0.86 | -0.15 | 0.74 | 0.10 | -0.18 | 0.16 | 0.67 | 0.08 |
| 1997 | -0.25 | 0.38 | 0.75 | 0.14 | -0.74 | -0.15 | 0.59 | 0.13 | -0.88 | -0.18 | 0.74 | 0.09 | -0.25 | 0.15 | 0.73 | 0.12 |
| 1998 | -0.30 | 0.35 | 0.75 | 0.15 | -0.77 | -0.09 | 0.63 | 0.14 | -0.88 | -0.13 | 0.74 | 0.10 | -0.26 | 0.12 | 0.74 | 0.14 |
| 1999 | -0.11 | 0.38 | 0.76 | 0.14 | -0.73 | -0.15 | 0.64 | 0.15 | -0.87 | -0.13 | 0.74 | 0.09 | -0.21 | 0.15 | 0.65 | 0.12 |
| 2001 | -0.29 | 0.40 | 0.75 | 0.12 | -0.76 | -0.13 | 0.59 | 0.14 | -0.87 | -0.12 | 0.74 | 0.10 | -0.24 | 0.16 | 0.74 | 0.13 |
| 2003 | -0.32 | 0.29 | 0.73 | 0.13 | -0.63 | -0.04 | 0.65 | 0.12 | -0.89 | -0.11 | 0.74 | 0.10 | -0.25 | 0.05 | 0.62 | 0.11 |
| 2004 | -0.25 | 0.43 | 0.79 | 0.14 | -0.84 | -0.20 | 0.68 | 0.14 | -0.98 | -0.17 | 0.74 | 0.10 | -0.25 | 0.20 | 0.65 | 0.11 |
| 2005 | -0.29 | 0.35 | 0.76 | 0.14 | -0.62 | -0.15 | 0.64 | 0.13 | -0.93 | -0.16 | 0.74 | 0.09 | -0.22 | 0.17 | 0.58 | 0.11 |
| 2007 | -0.23 | 0.28 | 0.73 | 0.13 | -0.58 | -0.07 | 0.70 | 0.13 | -0.88 | -0.11 | 0.74 | 0.09 | -0.30 | 0.16 | 0.54 | 0.12 |
| 2008 | -0.14 | 0.36 | 0.73 | 0.14 | -0.66 | -0.11 | 0.57 | 0.13 | -0.88 | -0.12 | 0.62 | 0.10 | -0.20 | 0.17 | 0.65 | 0.13 |
| 2009 | -0.24 | 0.35 | 0.77 | 0.14 | -0.65 | -0.08 | 0.68 | 0.14 | -0.92 | -0.10 | 0.74 | 0.11 | -0.27 | 0.11 | 0.60 | 0.12 |
| 2010 | -0.15 | 0.36 | 0.75 | 0.15 | -0.80 | -0.14 | 0.65 | 0.12 | -0.88 | -0.15 | 0.74 | 0.09 | -0.27 | 0.16 | 0.57 | 0.10 |
| 2011 | -0.31 | 0.32 | 0.73 | 0.15 | -0.56 | -0.07 | 0.68 | 0.13 | -0.87 | -0.12 | 0.74 | 0.10 | -0.30 | 0.12 | 0.49 | 0.10 |
| 2013 | -0.49 | 0.47 | 0.82 | 0.17 | -0.67 | -0.15 | 0.36 | 0.14 | -0.97 | -0.13 | 0.65 | 0.11 | -0.34 | 0.15 | 0.69 | 0.14 |
| 2014 | -0.43 | 0.51 | 0.84 | 0.16 | -0.82 | -0.18 | 0.37 | 0.14 | -0.97 | -0.16 | 0.66 | 0.10 | -0.33 | 0.20 | 0.75 | 0.15 |
| 2015 | -0.34 | 0.45 | 0.80 | 0.14 | -0.71 | -0.14 | 0.35 | 0.13 | -0.95 | -0.13 | 0.56 | 0.10 | -0.30 | 0.15 | 0.66 | 0.12 |
| 2016 | -0.39 | 0.47 | 0.83 | 0.16 | -0.64 | -0.15 | 0.35 | 0.14 | -0.93 | -0.14 | 0.49 | 0.10 | -0.32 | 0.16 | 0.64 | 0.14 |
| 2017 | -0.39 | 0.40 | 0.81 | 0.15 | -0.77 | -0.09 | 0.37 | 0.13 | -0.94 | -0.09 | 0.64 | 0.12 | -0.32 | 0.11 | 0.69 | 0.12 |
| 2018 | -0.33 | 0.42 | 0.80 | 0.16 | -0.79 | -0.12 | 0.38 | 0.15 | -0.96 | -0.12 | 0.44 | 0.11 | -0.27 | 0.17 | 0.70 | 0.14 |
| 2019 | -0.32 | 0.47 | 0.82 | 0.15 | -0.75 | -0.14 | 0.34 | 0.14 | -0.92 | -0.13 | 0.56 | 0.11 | -0.31 | 0.15 | 0.73 | 0.13 |
| 2020 | -0.55 | 0.46 | 0.85 | 0.16 | -0.79 | -0.14 | 0.39 | 0.14 | -0.96 | -0.13 | 0.44 | 0.11 | -0.32 | 0.15 | 0.76 | 0.14 |
| 2021 | -0.26 | 0.37 | 0.78 | 0.13 | -1.00 | -0.09 | 0.35 | 0.13 | -1.00 | -0.10 | 0.49 | 0.11 | -0.29 | 0.16 | 0.62 | 0.11 |

Table S4 Detailed statistics of the Ecological Evaluation Index threshold for the Imola during time span of 1991 to 2021

| **UTFVI** | **<0.000** | | **0.000-0.005** | | **0.005-0.010** | | **0.010-0.015** | | **0.015-0.020** | | **>0.020** | |
| --- | --- | --- | --- | --- | --- | --- | --- | --- | --- | --- | --- | --- |
| **EEI** | **Excellent** | | **Good** | | **Normal** | | **Bad** | | **Worse** | | **Worst** | |
|  | **Area (Km^2^)** | **Area (%)** | **Area**  **(Km^2^)** | **Area (%)** | **Area (Km^2^)** | **Area (%)** | **Area (Km^2^)** | **Area (%)** | **Area (Km^2^)** | **Area (%)** | **Area (Km^2^)** | **Area (%)** |
| **1991** | 105.77 | 51.60 | 11.16 | 5.44 | 6.97 | 3.40 | 2.75 | 1.34 | 14.06 | 6.86 | 64.26 | 31.35 |
| 1993 | 103.53 | 50.51 | 10.56 | 5.15 | 1.70 | 0.83 | 3.09 | 1.51 | 11.58 | 5.65 | 74.50 | 36.35 |
| 1994 | 108.48 | 52.92 | 7.42 | 3.62 | 4.48 | 2.19 | 9.41 | 4.59 | 8.57 | 4.18 | 66.61 | 32.50 |
| 1995 | 116.60 | 56.89 | 1.19 | 0.58 | 14.25 | 6.95 | 5.35 | 2.61 | 1.83 | 0.89 | 65.75 | 32.08 |
| 1996 | 111.92 | 54.60 | 5.96 | 2.91 | 3.45 | 1.68 | 13.93 | 6.79 | 4.14 | 2.02 | 65.57 | 31.99 |
| **1997** | 114.27 | 55.75 | 8.08 | 3.94 | 7.20 | 3.51 | 6.97 | 3.40 | 3.60 | 1.76 | 64.48 | 31.64 |
| 1998 | 100.39 | 48.98 | 6.95 | 3.39 | 0.06 | 0.03 | 11.30 | 5.51 | 3.05 | 1.49 | 83.22 | 40.60 |
| 1999 | 126.95 | 61.94 | 6.98 | 3.41 | 2.81 | 1.37 | 1.79 | 0.87 | 7.17 | 3.50 | 59.27 | 28.92 |
| 2001 | 109.31 | 53.33 | 2.34 | 1.14 | 3.44 | 1.68 | 8.81 | 4.30 | 2.93 | 1.43 | 78.14 | 38.12 |
| **2003** | 105.59 | 51.51 | 0.77 | 0.38 | 8.28 | 4.04 | 7.30 | 3.56 | 1.54 | 0.75 | 81.48 | 39.75 |
| 2004 | 113.58 | 55.42 | 3.75 | 1.83 | 7.45 | 3.63 | 3.47 | 1.69 | 6.44 | 3.14 | 70.28 | 34.29 |
| 2005 | 113.18 | 55.22 | 7.46 | 3.64 | 4.23 | 2.06 | 5.95 | 2.90 | 6.76 | 3.30 | 67.40 | 32.88 |
| 2007 | 113.75 | 55.50 | 5.76 | 2.81 | 3.82 | 1.86 | 11.88 | 5.80 | 3.12 | 1.52 | 66.63 | 32.51 |
| 2008 | 104.76 | 51.11 | 11.11 | 5.42 | 2.91 | 1.42 | 3.77 | 1.84 | 11.77 | 5.74 | 70.65 | 34.47 |
| **2009** | 112.11 | 54.70 | 2.81 | 1.37 | 10.46 | 5.11 | 1.78 | 0.87 | 4.92 | 2.90 | 69.90 | 34.10 |
| 2010 | 111.72 | 54.51 | 4.72 | 2.30 | 1.77 | 0.86 | 10.92 | 5.33 | 5.94 | 2.90 | 69.90 | 34.10 |
| 2011 | 101.38 | 49.46 | 9.46 | 4.61 | 3.78 | 1.84 | 5.81 | 2.83 | 7.87 | 3.84 | 76.67 | 37.41 |
| 2013 | 104.76 | 51.11 | 6.25 | 3.05 | 5.60 | 2.73 | 6.06 | 2.96 | 5.79 | 2.82 | 76.51 | 37.33 |
| 2014 | 111.43 | 54.37 | 6.30 | 3.07 | 6.11 | 2.98 | 6.22 | 3.04 | 6.05 | 2.95 | 68.86 | 33.59 |
| **2015** | 120.50 | 58.79 | 6.57 | 3.20 | 6.38 | 3.11 | 5.71 | 2.78 | 6.08 | 2.97 | 59.73 | 29.14 |
| 2016 | 101.63 | 49.58 | 7.34 | 3.58 | 7.40 | 3.61 | 7.30 | 3.56 | 7.88 | 3.84 | 73.41 | 35.81 |
| 2017 | 99.21 | 48.40 | 8.01 | 3.91 | 8.27 | 4.03 | 8.11 | 3.95 | 8.42 | 4.11 | 72.96 | 35.59 |
| 2018 | 106.71 | 52.06 | 6.79 | 3.31 | 6.65 | 3.25 | 6.84 | 3.34 | 7.00 | 3.42 | 70.97 | 34.62 |
| 2019 | 118.64 | 57.88 | 5.41 | 2.64 | 5.15 | 2.51 | 4.97 | 2.43 | 5.30 | 2.59 | 65.50 | 31.96 |
| 2020 | 99.27 | 48.43 | 4.83 | 2.36 | 4.65 | 2.27 | 4.79 | 2.34 | 5.26 | 2.57 | 86.17 | 42.04 |
| **2021** | 98.69 | 48.15 | 6.14 | 2.99 | 6.30 | 3.07 | 6.44 | 3.14 | 6.87 | 3.35 | 80.52 | 39.29 |
